# The Promise of AI for DILI Prediction

**DOI:** 10.3389/frai.2021.638410

**Published:** 2021-04-14

**Authors:** Andreu Vall, Yogesh Sabnis, Jiye Shi, Reiner Class, Sepp Hochreiter, Günter Klambauer

**Affiliations:** ^1^ LIT AI Lab, Johannes Kepler University Linz, Linz, Austria; ^2^ Institute for Machine Learning, Johannes Kepler University Linz, Linz, Austria; ^3^ UCB Biopharma SRL, Braine-l'Alleud, Belgium; ^4^ Institute of Advanced Research in Artificial Intelligence (IARAI), Vienna, Austria

**Keywords:** artificial intelligence, machine learning, neural networks, deep learning, drug-induced liver injury

## Abstract

Drug-induced liver injury (DILI) is a common reason for the withdrawal of a drug from the market. Early assessment of DILI risk is an essential part of drug development, but it is rendered challenging prior to clinical trials by the complex factors that give rise to liver damage. Artificial intelligence (AI) approaches, particularly those building on machine learning, range from random forests to more recent techniques such as deep learning, and provide tools that can analyze chemical compounds and accurately predict some of their properties based purely on their structure. This article reviews existing AI approaches to predicting DILI and elaborates on the challenges that arise from the as yet limited availability of data. Future directions are discussed focusing on rich data modalities, such as 3D spheroids, and the slow but steady increase in drugs annotated with DILI risk labels.

## 1 Introduction

DILI is a common cause of acute liver failure and one of the main reasons for failed clinical trials and for the withdrawal of drugs (Kaplowitz, 2004; Senior, 2007). Hepatotoxicity can be anticipated in some cases. For example, acetaminophen (also known as paracetamol) is known to damage the liver when used beyond the recommended dose (Lancaster et al., 2015). However, other DILI events are considered idiosyncratic; that is, they are rare and difficult to predict (Hoofnagle and Björnsson, 2019). Research directions such as the investigation of reliable biomarkers (Wang et al., 2009) and the development of AI methods (Przybylak and Cronin, 2012; Chen et al., 2014) aim to improve the understanding of DILI mechanisms and to anticipate hepatotoxicity early in the drug development process.

We elaborate on the latter by providing a review of the state of the art in AI for DILI prediction, focusing particularly on approaches based on machine learning (ML). From an ML perspective, DILI prediction is generally cast as a supervised learning problem (Murphy, 2012, Section 1.1.1). Let us consider a chemical compound *c*, the hepatotoxicity risk of which can be represented by a scalar $y_{c}$. Let us further assume that relevant properties of the compound can be characterized by a vector $x_{c}$. Generally, ML approaches to predicting DILI model the relationship between $y_{c}$ and $x_{c}$ by means of an approximating function *f* such that $y_{c}\approx f\left(x_{c}\right)$ holds for the largest possible domain of compounds. This approach is generally termed “*in silico*” due to its computer-based nature.

The remainder of this review article is divided into four sections. Section 2 presents the main datasets that list compounds and their hepatotoxicity risk ($y_{c}$); Section 3 discusses various data modalities used to describe the chemical characteristics of compounds ($x_{c}$); Section 4 examines the ML methods proposed in the literature to model the relationship between $y_{c}$ and $x_{c}$ (*f*). Some studies reviewed here feature in more than one of these three sections. Section 5 concludes this contribution with a discussion. For quick reference, we provide an index of the studies reviewed with a summary of their main characteristics (Table 1, Supplementary Material).

**TABLE 1:** Scientific studies considered in this review ordered alphabetically by author name (disregarding the year of publication).

Study	Topic	DILI annotations	Data modalities	Predictive models
Ai et al. (2018)	ML	Training dataset derived from Liew et al. (2011) and Zhu and Kruhlak (2014). LTKB dataset (Chen et al., 2011) as validation set.	PaDEL-Descriptor v2.21 molecular descriptors.	Random forests; Support vector machines; XGBoost.
Björnsson and Hoofnagle (2016)	Dataset	Derived from the LiverTox website (Hoofnagle et al., 2013).	—	—
Chalasani et al. (2015)	Dataset	Collected in the US as part of the efforts of the drug-induced liver injury network (DILIN). Includes causality assessments.	—	—
Chen et al. (2011)	Dataset	Derived from FDA-approved drug labels.	—	—
Chen et al. (2013a)	ML	LTKB dataset (Chen et al., 2011) and dataset published by Greene et al. (2010).	Daily dose and lipophilicity provided by Pipeline Pilot 8.0.	Rules.
Chen et al. (2013b)	ML	Training dataset is an extension of the LTKB dataset (Chen et al., 2011). Test on the datasets published by Greene et al. (2010) and Xu et al. (2008).	Mold^2^ molecular descriptors.	Decision forests (Tong et al., 2003).
Chen et al. (2014)	ML review	—	—	—
Chen et al. (2016)	Dataset	Extension of Chen et al. (2011) with additional drugs and a subsequent verification step. Verification relying on Suzuki et al. (2010), Hoofnagle et al. (2013) and Chalasani et al. (2015).	—	—
Chierici et al. (2020)	ML	Binary labels provided by the CMap Drug Safety Challenge 2018.	Gene expression data provided by the CMap Drug Safety Challenge 2018.	Random forests; Shallow neural networks; Deep neural networks.
Clevert et al. (2012)	ML	LTKB dataset (Chen et al., 2011).	Gene expression data from the Japanese Toxicogenomics Project.	Support vector machines.
Cruz-Monteagudo et al. (2008)	ML	Custom selection of positives previously reported by Li (2002) and negatives selected from a drug compendium.	RDF and other molecular descriptors computed with Dragon.	Linear discriminant analysis; Neural networks; One-rule classifiers.
Ekins et al. (2010)	ML	Extension of Xu et al. (2008).	Extended connectivity fingerprints.	Naive Bayes classifiers.
Feng et al. (2019)	ML	*In vivo* assays on rats.	Gene expression data corresponding to diverse hepatotoxicity categories.	Support vector machines; Shallow neural networks.
Garside et al. (2014)	*In vitro*	Derived from drug labels and literature.	Measurements from high content fluorescence microscopy.	Rules; Hierarchical cluster analysis.
Greene et al. (2010)	Dataset; ML	Own training dataset derived from literature. Test dataset overlapping with Xu et al. (2008).	Chemical structures exported as an SD file.	Structural alerts.
He et al. (2019)	Dataset; ML	Training on a dataset comprising the LTKB (Chen et al., 2011), Livertox (Björnsson and Hoofnagle, 2016), and DILIrank (Chen et al., 2016) datasets, extended with additional compounds. Test on a dataset comprising the datasets used by Ai et al. (2018), Zhang et al. (2016), Kotsampasakou et al. (2017).	Marvin molecular descriptors.	Ensemble of classifiers, including naive Bayes, *k*-nearest neighbors, random forest, and an off-the-shelf deep learning solution.
Hong et al. (2017)	ML	Subset of the DILIrank dataset (Chen et al., 2016).	Mold^2^ molecular descriptors.	Decision forests (Tong et al., 2003).
Hoofnagle and Björnsson (2019)	Review	—	—	—
Huang et al. (2010)	ML	*In vivo* assays on rats.	Genomic indicators from the blood.	Random forests; Support vector machines; *k*-nearest neighbors; Nearest centroid.
Kaplowitz (2004)	Review	—	—	—
Khadka et al. (2019)	ML; AOP	DILIrank dataset (Chen et al., 2016).	AOP-supported selection and integration of various high throughput predictors.	Logistic regression.
Kohonen et al. (2017)	ML	Derived from the NCI-60 DTP human tumor cell line (Shoemaker, 2006).	Gene expression data from the CMap dataset (Lamb et al., 2006).	Ad hoc probabilistic model.
Lamb et al. (2006)	Dataset	—	Gene expression data.	—
Li et al. (2020a)	ML	DILIst dataset (Thakkar et al., 2020).	Drug-induced transcriptome profiles curated from the NIH LINCS L1000 dataset (Subramanian et al., 2017).	Deep neural networks.
Li et al. (2020b)	ML	DILIst dataset (Thakkar et al., 2020).	Mold^2^ molecular descriptors.	Deep neural network ensembling classifiers, including logistic regression, *k*-nearest neighbors, support vector machine, random forest, and XGBoost.
Liew et al. (2011)	Dataset; ML	Drugs collected from the FDA Orange Book. Annotations derived from drug compendia and literature.	PaDEL-Descriptor v2.0 molecular descriptors.	Ensemble of many instances of *k*-nearest neighbors, support vector machines, and naive Bayes classifiers.
Liu et al. (2011)	ML	LTKB dataset (Chen et al., 2011) and datasets published by Greene et al. (2010) and O’Brien et al. (2006).	Functional class fingerprints (FCFP_6) provided by Pipeline Pilot 8.0.	Naive Bayes classifiers.
Minerali et al. (2020)	ML	DILIrank (Chen et al., 2016), BDDCS (Benet et al., 2011) and “Withdrawn” datasets (Siramshetty et al., 2016), and also from literature (Hong et al., 2017; Aleo et al., 2020; Williams et al., 2020).	Extended-connectivity fingerprints (ECFP_6).	Random forests; *k*-nearest neighbors; Support vector machines; Naive Bayes classifiers; AdaBoosted decision trees; Deep learning.
Proctor et al. (2017)	*In vitro*	Derived from drug labels and literature following the classification proposed by Garside et al. (2014).	3D human liver microtissues; Plated 2D primary human hepatocytes.	Rules based on thresholds optimizing the ROC curve.
Przybylak and Cronin (2012)	ML review	—	—	—
Puri (2020)	ML	Japanese Toxicogenomics Project.	Histopathology whole slide images.	Standard computer vision; Computer vision built with automated ML.
Sakatis et al. (2012)	Dataset; ML	Derived from drug labels and reports.	Clinical dose, GSH adduct formation, and P450 MDI.	Decision trees.
Suzuki et al. (2010)	Dataset	From DILI regulatory agencies and literature. Includes frequency of reports.	—	—
Thakkar et al. (2020)	Dataset	Derived from Chen et al. (2016), Björnsson and Hoofnagle (2016), Suzuki et al. (2010), Greene et al. (2010), and Zhu and Kruhlak (2014).	—	—
Uehara et al. (2010)	Dataset	*In vivo* assays on rats.	Genomic biomarkers predictive of the toxicity of chemicals.	—
Vorrink et al. (2018)	*In vitro*	Derived from regulatory classifications and literature.	3D spheroid cultures.	Rules.
Wang et al. (2009)	Biological study	—	—	—
Wang et al. (2019)	ML	Subset of the DILIrank dataset (Chen et al., 2016).	PaDEL-Descriptor v2.21 molecular descriptors.	Ensemble of classifiers, including logistic regression, support vector machines, random forests, and multiple gradient boosting decision tree approaches.
Williams et al. (2020)	ML	Derived from the LTKB (Chen et al., 2011) and DILIrank (Chen et al., 2016) datasets, derived from the literature, and in-house annotation as described by Proctor et al. (2017).	C_max_, cLogP, bioactivation, HepG2/C3A spheroid cytotoxicity, mitochondrial toxicity, BSEP inhibition, THP-1 cytotoxicity, cytotoxicity EC_50_ values.	Bayesian ordered logit model.
Xu et al. (2008)	Dataset; *In vitro*; ML	Derived from clinical data, drug labels, reports, and preclinical animal toxicology data.	Descriptors derived from human hepatocyte 2D images.	Standard computer vision; Rules; Random forests.
Xu et al. (2015)	ML	Datasets by Xu et al. (2008), Greene et al. (2010), Liew et al. (2011), and Chen et al. (2013b), and a combined dataset integrating the datasets by Xu et al. (2008), Greene et al. (2010), and Chen et al. (2013b).	Molecular structure directly.	Undirected graph recursive neural networks proposed by Lusci et al. (2013).
Zhu and Kruhlak (2014)	Dataset	Dataset derived from FAERS, including frequency of reports. Additional calibration dataset extending Suzuki et al. (2010).	—	Rules based on setting a threshold that optimizes the calibration ROC curve.
Zhu et al. (2014)	ML	Dataset by Xu et al. (2008).	Molecular descriptors computed with Chemistry Development Kit (v.1.4.13), Dragon (v.5.5), Molecular Operating Environment (v.2009.10), and MayaChemTools; Descriptors derived from human hepatocyte 2D images (Xu et al., 2008); Combination of both.	Random forests.

The second column lists one or several keywords identifying the general topic of the study. Columns three, four and five summarize the relation (if any) of each study to Sections 2, 3, and 4, respectively.

## 2 Drug-Induced Liver Injury Annotations

We describe the main DILI annotation datasets that are publicly available, more specifically, categorizations of drugs based on their DILI risk in humans under medication (Chen et al., 2016). DILI annotations are necessary not only to train supervised machine learning models, but also to evaluate the performance of any predictive model (even of simple models such as structural alerts).

### 2.1 Dataset by Xu et al. (2008)

Xu et al. (2008) assembled a dataset of DILI annotations to validate a proposed cellular-imaging-based testing strategy. The dataset contained 344 drugs and chemicals with annotations derived from verified clinical hepatotoxicity data, drug labels, reports, and preclinical animal toxicology data. The annotation scheme distinguished between DILI “positive” and DILI “negative.” Ekins et al. (2010) used the dataset by Xu et al. (2008) to train naive Bayes classifiers. These were then tested on an additional dataset of 237 compounds which had already been curated by Xu et al. together with the initial dataset, but had not been available for *in vitro* testing at that time. Greene et al. (2010) extended the dataset by Xu et al. (2008) to a total of 626 compounds in total. Furthermore, they modified the annotation scheme, splitting the negative cases into “no evidence” and “weak evidence” of hepatotoxicity.^1^ The dataset was used to evaluate the performance of a structural-alert-based DILI prediction model.

### 2.2 Dataset by Suzuki et al. (2010)

Suzuki et al. (2010) collected a dataset of 319 drugs associated with hepatotoxicity. The sources of information were DILI registries from Spain, Sweden, and the US, studies of acute liver failure in these countries, and other published literature. The collection was supplemented with the frequency of liver adverse events reported in the World Health Organization VigiBase database (Lindquist, 2008). Zhu and Kruhlak (2014) took a subset of 177 drugs from this dataset and extended it with 105 drugs presumed to be DILI negatives based on the absence of warnings on PubMed^2^ and the US Food and Drug Administration (FDA) MedWatch (Kessler et al., 1993) after more than five years on the market. This dataset was used as a calibration set to develop a simple DILI prediction model. Zhu and Kruhlak also constructed another dataset of DILI annotations by querying the FDA Adverse Event Reporting System (FAERS) database.^3^

### 2.3 LiverTox Dataset

The LiverTox dataset provides information on drug, dietary supplement and herbal-induced liver injury (Hoofnagle et al., 2013). The main body of the dataset is a collection of drug hepatotoxicity records, but additional resources are provided to support the critical study of DILI. Importantly, causality assessment instruments, such as the Roussel Uclaf Causality Assessment Method (RUCAM) (Danan and Teschke, 2019) and the Drug Induced-Liver Injury Network Causality Process (Fontana et al., 2009), are presented and discussed.

Björnsson and Hoofnagle (2016) conducted a critical analysis of 671 distinct drugs reported in the LiverTox dataset, and categorized them according to the number of published reports of idiosyncratic liver injury. Case reports in specific categories were reanalyzed using RUCAM. Chen et al. (2016) built on this categorization to improve the accuracy of drug-label-based annotations by incorporating evidence of causality. Thakkar et al. (2020) also used it but to increase the size of their dataset.

### 2.4 Food and Drug Administration Datasets

Chen et al. (2011) compiled the so-called “LTKB” dataset, which was based exclusively on drug labels retrieved from DailyMed.^4^ The rationale behind this approach was that drug labels implicitly integrate information on causality, incidence, and severity from trials, existing literature, and reports. Furthermore, drug labels must be reviewed regularly, and thus the annotations can be kept up to date. A classification by concern level was developed using three labels: “most-DILI,” “less-DILI,” and “no-DILI” concern.

The LTKB dataset, which consisted of 287 drugs, was later extended by refining the classification scheme and including additional drugs (Chen et al., 2016). The new classification scheme included whether drugs had been verified as the cause of DILI in humans, and assigned four risk levels: three corresponded to those of the LTKB dataset, but with verification, while the fourth one covered drugs for which the DILI annotation was ambiguous. The verification relied on published studies which had focused on causality assessment (Suzuki et al., 2010; Hoofnagle et al., 2013; Chalasani et al., 2015). More drugs were included in the dataset by considering drugs approved by the FDA over a longer time frame. This new version of the dataset, dubbed “DILIrank,” included 1,036 marketed drugs, 254 of which were annotated as being ambiguous.

Thakkar et al. (2020) further extended the DILIrank dataset by carefully incorporating drug annotations from previously published datasets (Greene et al., 2010; Suzuki et al., 2010; Zhu and Kruhlak, 2014; Björnsson and Hoofnagle, 2016). The authors took the DILIrank dataset as a basis, and drugs from the other datasets were incorporated when sufficient agreement between annotations was found. The extended dataset, named “DILIst,” contains 1,279 drugs classified according to a binary scheme.

### 2.5 Other Datasets

Other DILI annotation datasets exist, but have not been used as routinely in subsequent studies. Cruz-Monteagudo et al. (2008) curated a dataset of 74 drugs by combining DILI positives from a previously published collection (Li, 2002) with drugs manually selected as not having an association with hepatotoxicity according to a drug compendium. Liew et al. (2011) assembled a list of 1,274 drugs from the FDA Orange Book^5^ with DILI annotations derived from the Micromedex Health Care Series and with additional compounds from literature reports. Other studies compiled custom datasets with a few hundred datapoints and annotations based on the existence or absence of hepatotoxicity reports, on previous literature, and on expert opinion (Sakatis et al., 2012; Garside et al., 2014; Proctor et al., 2017; Vorrink et al., 2018).

## 3 Data Modalities

Chemical compounds can be described using various data modalities, such as chemical structure, gene expression profiles, and cell and tissue images. ML methods are generally not limited to a specific modality when producing a DILI prediction model. For this reason, we provide an overview of the most common data modalities used in DILI prediction, regardless of the specific ML method considered.

### 3.1 Chemical Structure

Analysis of the chemical structure of compounds is frequently used and has the notable advantage of naturally always being available. This approach, generally referred to as quantitative structure–activity relationship (QSAR) modeling, is widely applied in chemoinformatics, also beyond the prediction of DILI risk. A well-established pipeline first computes feature vectors, typically called “molecular descriptors,” which encode structural properties of the compounds. These descriptors are then passed to a ML model of choice. Various kinds of descriptors have been proposed, ranging from simple characteristics, such as molecular weight and number of carbon atoms, to more sophisticated encodings, which are typically called “molecular fingerprints” (Morgan, 1965; Quist, 2006; Rogers and Hahn, 2010).

A number of software implementations exist to compute standard molecular descriptors. In many cases, authors specify their set of descriptors by referring to the software implementation employed. Cruz-Monteagudo et al. (2008) computed radial-distribution-function descriptors with the DRAGON software (Mauri et al., 2006). Liu et al. (2011) employed functional class fingerprints (FCFP_6) provided by the Pipeline Pilot 8.0 software. Chen et al. (2013a) also employed Pipeline Pilot 8.0, but in this case to estimate drug lipophilicity. Liew et al. (2011), Ai et al. (2018) and Wang et al. (2019) used descriptors provided by the PaDel-Descriptor software (Yap, 2011). Chen et al. (2013b) and Hong et al. (2017) used Mold^2^ descriptors (Hong et al., 2008). He et al. (2019) computed descriptors with the Marvin software.^6^

Some ML methods can directly process the chemical structure of compounds. Xu et al. (2015) followed the approach proposed by Lusci et al. (2013), where molecular graphs are passed directly to a recursive neural network. For general (not DILI-specific) target prediction tasks, Mayr et al. (2018) used two such ML methods: one processed SMILES strings (Weininger, 1988) by using long short-term memory recurrent neural networks (Hochreiter and Schmidhuber, 1997), and the other processed molecular graphs with graph convolutional neural networks (Duvenaud et al., 2015).

### 3.2 Gene Expression

To complement and improve chemical-structure-based models, additional information in the form of gene expression data can be useful (Martin et al., 2002; Klambauer et al., 2015). In this context, there has been notable work investigating the advantage of considering genomic biomarkers for DILI prediction. Huang et al. (2010) successfully used genomic indicators to predict acetaminophen-induced liver injury. Clevert et al. (2012) proposed a pipeline with suitable pre-processing steps for DILI prediction using data from the Japanese Toxicogenomics Project (TGP) (Uehara et al., 2010). Feng et al. (2019) also used data from the TGP and proposed a DILI prediction model based on a feed-forward neural network. Kohonen et al. (2017) predicted DILI using a model which leveraged both the CMap dataset and the NCI60 cell line screen (Shoemaker, 2006). Chierici et al. (2020) utilized a subset of the CMap dataset of two specific cell lines, as provided in the CMap Drug Safety Challenge 2018,^7^ although the authors found that these data were insufficient for DILI prediction. Li et al. (2020a) proposed a DILI prediction model based on a deep neural network, which takes as input transcriptomic profiles of human cell lines derived from the LINCS L1000 dataset (Subramanian et al., 2017).

### 3.3 *In vitro* and Imaging Assays

Xu et al. (2008) developed an *in vitro* testing strategy for DILI prediction based on features measured by high-content cellular imaging in primary human hepatocyte cultures. Of a total of eight features extracted using standard computer vision algorithms, the features “mitochondrial damage,” “oxidative stress,” and “intracellular glutathione” were found to be the most important for DILI prediction. Zhu et al. (2014) further investigated the predictive power of this imaging data and compared it to using molecular descriptors alone, or a combination of imaging data and molecular descriptors.

Garside et al. (2014) investigated a number of previously proposed hepatotoxicity prediction assays, which utilized either HepG2 cells, HepG2 cells in the presence of rat S9 fraction, or isolated human hepatocytes. Images were acquired by means of high-content fluorescence microscopy.

Puri (2020) used histopathology whole-slide images to train a computer vision system with automated ML. The system was able to predict whether images corresponded to 1 of 10 drugs, and this was interpreted as an ability to discriminate between DILI injury patterns. However, the usefulness of this system is limited due to the absence of DILI risk predictions and the reduced chemical space considered.

3D cell cultures have gained attention over 2D cell cultures (Huh et al., 2011): they grow longer, are more stable and reflect actual liver responses more accurately, therefore having higher predictive power (Messner et al., 2013; Proctor et al., 2017; Vorrink et al., 2018). Combining these with physicochemical and exposure variables, and with data from other *in vitro* assays, Williams et al. (2020) considered the outcome of an HepG2/C3A spheroid cytotoxicity assay for their DILI prediction model. 3D cultures can not only be employed as purely biological assays; they are also compatible with imaging technologies.^8^

To our knowledge, neither 2D nor 3D imaging technologies have to date been used in combination with advanced computer vision techniques based on deep learning.

## 4 Predictive Models

We review the main DILI prediction models proposed to date. For the sake of clarity, we categorize them into three main categories: rules and knowledge-based systems, shallow ML methods, and deep learning methods. Rules and knowledge-based systems rely on explicitly coded decision rules. This is in contrast to ML methods, the decision mechanisms of which are implicitly defined and obtained by means of optimization techniques. Examples of shallow ML methods are naive Bayes classifiers and random forests. Deep learning methods are ML methods based on the so-called “deep” neural networks, which are considered to be those with at least two hidden layers (Delalleau and Bengio, 2011; Maas et al., 2012).

### 4.1 Rules and Knowledge-Based Systems

DILI risk can be predicted from *in vitro* assays and simple decision rules. Garside et al. (2014) and Vorrink et al. (2018) considered that an *in vitro* assay yielded a positive prediction when its outcome differed significantly from the control value, going on to evaluate the predictive power of this decision rule by means of the usual classification metrics. Similarly, Proctor et al. (2017) built a classifier by selecting a threshold for each assay such that the resulting ROC curves were optimized.

Greene et al. (2010) proposed a predictive model based on structural alerts, that is, a system based on expert knowledge of which chemical substructures are related to hepatotoxicity. Chen et al. (2013a) analyzed the effect of daily dose and lipophilicity on hepatotoxicity. They found the “rule-of-two,” by which drugs with daily doses ≥100 mg and logP≥3 were likely to be hepatoxic. Zhu and Kruhlak (2014) developed classifiers based on thresholds for signals derived from counts in the FAERS database, optimizing the ROC curve on a calibration set.

### 4.2 Shallow Machine Learning Methods

Xu et al. (2008) explored rule-based systems but achieved the best predictive performance by processing their proposed imaging features with a random forests model (Ho, 1995; Breiman, 2001). Subsequent work by Zhu et al. (2014) also used random forests, in this case within a 5-fold cross-validation model selection scheme. Chen et al. (2013b) built predictive models for DILI using decision forests (Tong et al., 2003) with 2,000 repetitions of 10-fold cross-validation for model selection. Decision forests differ from random forests in that each tree uses the whole training set and an explicit selection of features. This work was based on the LTKB dataset, but Hong et al. (2017) extended it to the DILIrank dataset.

Ekins et al. (2010) used a naive Bayes classifier with various types of molecular fingerprints. Similarly, Liu et al. (2011) used naive Bayes classifiers with molecular fingerprints, but in this case an independent classifier was trained for each of 13 hepatotoxic side effects. A final classifier was derived by means of a consensus strategy. Kohonen et al. (2017) introduced an ad hoc probabilistic model for the toxicogenomic space based on the latent Dirichlet allocation model (Blei et al., 2003). Williams et al. (2020) proposed a probabilistic ordered logit model that distinguishes between three increasing levels of DILI risk.

Cruz-Monteagudo et al. (2008) provided an evaluation of DILI prediction models that are based on various ML methods, namely a classifier based on a single attribute (Holte, 1993), linear discriminant analysis, and neural networks. Liew et al. (2011) proposed a DILI prediction model that consists of an ensemble of 617 base classifiers. These base classifiers were numerous instances of *k*-nearest neighbors classifiers, naive Bayes classifiers, and support vector machines (Cortes and Vapnik, 1995), each of which trained with a different subset of molecular descriptors. The final ensemble model was selected using 5-fold cross-validation.

### 4.3 Deep Learning Methods

Despite the rise of deep learning over the last decade, only few approaches to DILI prediction are based on this technology. Lusci et al. (2013) introduced undirected graph recursive neural networks (UG-RNNs) to predict the aqueous solubility of drug-like molecules. UG-RNNs bridge the gap between molecules (described as undirected cyclic graphs) and recursive neural networks (expecting directed acyclic graphs). In hindsight, this method can be regarded as a precursor of the now successful family of convolutional neural networks for graph structures (Duvenaud et al., 2015). Xu et al. (2015) employed UG-RNNs for DILI prediction. They also evaluated feed-forward neural networks of various depths, using PaDEL or Mold^2^ descriptors as input. UG-RNNs were found to perform best, followed by deep feed-forward neural networks and then by shallow feed-forward neural networks.

He et al. (2019) proposed an ensemble DILI prediction model which included deep neural networks. However, the only information provided about the deep neural network component was that it was implemented within the Deeplearning4j^9^ framework. Chierici et al. (2020) investigated deep learning architectures for DILI prediction using toxicogenomics data. They compared deep and shallow neural networks and random forests classifiers in terms of performance. The conclusion of this work was ambiguous. The authors claimed that the dataset used, published in the context of the CMap Drug Safety Challenge 2018, was not rich enough to build predictive models for DILI.

Li et al. (2020a) proposed a DILI prediction model consisting of a deep neural network which leveraged transcriptomic profiles of human cell lines. It outperformed other shallow ML methods, namely *k*-nearest neighbors, support vector machines and random forests. In another study, the same authors proposed an ensemble prediction model where a deep neural network aggregated the DILI risk probabilities predicted by roughly 500 base classifiers (Li et al., 2020b). The base classifiers were numerous instances of logistic regression, *k*-nearest neighbors, support vector machines and random forests, and each used Mold^2^ descriptors as input. The ensemble model outperformed the individual base classifiers, and it also outperformed a deep neural network which directly used Mold^2^ descriptors as input.

## 5 Discussion

DILI is a common cause of liver failure, failed clinical trials, and withdrawal of drugs from the market. This work has reviewed the state of the art of AI approaches to predicting DILI, focusing on the approaches that are based on ML methods. Below, we discuss open challenges and future research directions.

ML approaches to DILI prediction are limited by the availability of DILI annotations. As mentioned in Section 2, the DILIst dataset, which is one of the largest and most comprehensive DILI annotation dataset, comprises only 1,279 drugs (Thakkar et al., 2020). The DILIst dataset is orders of magnitude smaller than benchmarking datasets in drug discovery (Mayr et al., 2018), and even smaller compared to benchmarking datasets in other AI application domains such as computer vision (Deng et al., 2009) and natural language processing (Vaswani et al., 2017), which can contain millions of data points. From an ML perspective this is critical, especially for deep learning methods, the remarkable success of which is presumably due to the access to large amounts of data (Goodfellow et al., 2016, Section 1.2.2). However, this limitation is a consequence of the also limited number of available drugs overall. Indeed, the latest release of DrugBank (version 5.1.8) lists a total of 14,331 drugs, only 2,677 of which are approved small molecule drugs.^10^

Despite the efforts of the research community to better understand its causes, DILI cannot yet be fully explained. Particularly for the cases considered idiosyncratic, which are very infrequent and lack a clear dose-response relationship (Björnsson and Hoofnagle, 2016), it can be challenging to annotate the DILI risk unambiguously. ML methods, and especially deep learning, excel at uncovering salient patterns from data. However, even if they exist, patterns obscured by noisy annotations can be difficult to reveal. Some of the reviewed studies carried out permutation tests, mostly *y*-randomization (Rücker et al., 2007), to verify that the proposed models were indeed superior to random ones (Liew et al., 2011; Zhu et al., 2014; He et al., 2019). Research in the domain of aleatoric uncertainty estimation (Brando et al., 2019) may provide further help in identifying such effects.

Another consequence of the complexity of DILI is the variety of published risk classification schemes. These are generally ordered from less to more DILI risk, and are divided into two or more severity levels. The existence of different classification schemes is not problematic in itself, because it is possible to employ binary and multi-class prediction models depending on the classification scheme under consideration. Even various classification schemes can be leveraged simultaneously by means of multitask learning (Mayr et al., 2018). However, DILI annotations reported in various datasets are not always reconcilable with each other (Thakkar et al., 2020), which is clearly problematic for model development. An effort towards standardization of classification schemes and annotations will be essential to the development of ML methods for DILI prediction.

The standardization of training and test splits is also necessary. Consider the following example. Li et al. (2020a) and Li et al. (2020b) both used the DILIst dataset (Thakkar et al., 2020) to build predictive models. However, Li et al. (2020a) split the dataset according to the availability of transcriptomic profiles, while Li et al. (2020b) split it according to the initial year when the FDA approved the drugs. The predictive performance results obtained on these different splits cannot be compared. Consider another example. Li et al. (2020b) provided further predictive performance results using other datasets as the independent test set. Among others, they used the dataset published by Greene et al. (2010), but subtracted the drugs that also occurred in their training set in order to obtain a truly independent test set. This operation reduced the dataset originally published by Greene et al. (2010) from 209 DILI positives and 111 DILI negatives to only 52 DILI positives and 28 DILI negatives. The performance results obtained on the reduced version of the dataset cannot be compared to other results obtained on the original dataset.

Taken together, a fair comparison of the numerous DILI prediction models proposed to date requires the standardization of datasets, also in terms of fixed training and test splits. The FDA is leading this endeavor, with a continuous line of studies consolidating DILI classification schemes and extending the list of annotated drugs available (Chen et al., 2011; Chen et al., 2016; Thakkar et al., 2020).

Several of the DILI prediction models reviewed are based exclusively on exploiting the chemical structure of compounds. While the natural availability of structural information makes this approach very flexible, it can also fall short. Some of the adverse reactions considered idiosyncratic may be undetectable from the chemical structure alone, but might be predictable if genomic data is also considered. In this context, the reviewed studies focusing on the exploitation of gene expression data (Huang et al., 2010; Clevert et al., 2012; Kohonen et al., 2017; Chierici et al., 2020) are particularly relevant to increase the understanding of the possible dependence of idiosyncratic DILI on genetic host factors (Stephens and Andrade, 2020). Complementarily, Khadka et al. (2019) investigated the potential of the adverse outcome pathway (AOP) framework to improve the selection and integration of various high throughput predictors relevant to DILI prediction. The authors focused on DILI risk assessment, but the AOP framework can address other types of chemical safety assessment (Wittwehr et al., 2017).

We also see an opportunity for improvement in the exploitation of *in vitro* 2D and 3D imaging data, namely by using advanced deep-learning-based computer vision methods. Computer vision has progressed remarkably fast in recent years, also in the domain of biomedical imaging (Esteva et al., 2017). However, the image-based predictive models for DILI proposed thus far generally rely on standard computer vision techniques (Xu et al., 2008; Zhu et al., 2014). Puri (2020) used an automated ML engine to train a deep learning classifier for histopathology images, but no details of the model architecture were shared. The number of drugs with 2D, and especially 3D, imaging data available is as yet limited. The acquisition of imaging data will be necessary to enable progress in this area.

Returning to DILI prediction models that are based on the chemical structure of compounds, while we find that deep learning methods have been proposed, they neither show an outstanding improvement in predictive performance (He et al., 2019; Minerali et al., 2020), nor have they been able to replace *in vitro* or *in vivo* tests. Generally, the proposed deep learning methods were based on processing pre-calculated molecular descriptors. Only Xu et al. (2015) considered and end-to-end approach, building on the UG-RNN method (Lusci et al., 2013), which was able to directly process the chemical structure of compounds and implicitly derive suitable molecular representations. In this regard, recent advances in graph convolutional neural networks (Gilmer et al., 2017; Li et al., 2019)—which are also end-to-end—should be investigated for DILI prediction.

Overall, we envision that new, more powerful deep learning methods for DILI prediction will be proposed in the near future, both in the domains of imaging and graph convolutional neural networks. Predictive models with high predictive performance may become not only screening tools, but potentially “virtual assays” (Mayr et al., 2018) able to replace *in vitro* and *in vivo* tests.

## Footnotes

1
Greene et al. (2010) considered a fourth category for animal hepatotoxicity not tested in humans, but we omit it here.

2
https://pubmed.ncbi.nlm.nih.gov [accessed on January 18, 2021].

3
https://www.fda.gov/drugs/surveillance/questions-and-answers-fdas-adverse-event-reporting-system-faers [accessed on January 18, 2021].

4
https://dailymed.nlm.nih.gov/dailymed [accessed on January 18, 2021].

5
https://www.accessdata.fda.gov/scripts/cder/ob/index.cfm [accessed on January 18, 2021].

6
https://chemaxon.com/products/marvin [accessed on January 18, 2021].

7
http://camda2018.bioinf.jku.at/doku.php/contest_dataset\#cmap_drug_safety_challenge [accessed on January 18, 2021].

8
https://www.cyprotex.com/toxicology/3d-microtissue-models/3d-imaging-of-microtissues [accessed on January 18, 2021].

9
https://deeplearning4j.org [accessed on January 18, 2021].

10
https://go.drugbank.com/about [accessed on January 18, 2021].
